# Exploring the role of infectious pathogens in Alzheimer’s disease neuroinflammation

**DOI:** 10.3389/fcimb.2026.1919289

**Published:** 2026-09-21

**Authors:** Salvatore Vaiasicca, Gianmarco Mangiaterra, Barbara Citterio, Giorgia Piccioni, Fiorenza Orlando, Belinda Giorgetti, Laura Cianfruglia, Robertina Giacconi, Stefania Fumarola, Maurizio Cardelli, Leonardo Biscetti, Paolo Pelliccioni, Tiziana Casoli, Giuseppe Pelliccioni

**Affiliations:** 1 Center of Biogerontology, IRCCS INRCA, Ancona, Italy; 2 Department of Biomolecular Sciences, University of Urbino Carlo Bo, Biotechnology section, Fano, Italy; 3 Experimental Animal Models for Aging Unit, Scientific Technological Area, IRCCS INRCA, Falconara, Marittima, Italy; 4 Neurology Unit, IRCCS INRCA, Ancona, Italy; 5 Ophthalmology Department, AST Pesaro Urbino, Pesaro, Italy

**Keywords:** Alzheimer’s disease, blood-brain barrier, infectious pathogens, microbiota-gut-brain axis, neuroinflammation

## Abstract

Alzheimer’s disease (AD) is the most common neurodegenerative disorder in older adults, characterized by progressive cognitive decline that ultimately leads to severe dementia. The primary pathophysiological hallmarks of AD include the accumulation of amyloid-β plaques, neurofibrillary tangles composed of hyperphosphorylated tau protein, synaptic dysfunction, and pronounced neuroinflammation. Although the exact relationship between neuroinflammation and AD pathogenesis remains incompletely understood, neuroinflammation is consistently recognized as an early and sustained feature of the disease. Accumulating evidence suggests that microbial infections, such as herpes simplex virus type 1, varicella-zoster virus, and *Porphyromonas gingivalis*, may play a role in the etiology and progression of AD and its associated neuroinflammatory processes. The gut microbiota may also contribute to AD pathogenesis through a complex communication network known as the microbiota–gut–brain axis. Dysbiosis, defined as an imbalance in the gut microbial community, may promote AD development and progression by enhancing neuroinflammation and producing microbial-derived toxic metabolites that can impair brain function. Neuroinflammation, in turn, contributes to the disruption of blood–brain barrier integrity, thereby facilitating the infiltration of peripheral immune cells and pathogens into the central nervous system. Importantly, chronic infections may lead to sustained immune activation, establishing a vicious cycle that further amplifies neuroinflammation, thus potentially accelerating AD progression. Emerging evidence highlights the interplay between systemic infections, immune dysregulation, and neurodegenerative processes, suggesting that pathogen-driven inflammation may represent a modifiable risk factor and a potential therapeutic target in AD. In this review, we examine the putative role of infectious pathogens in the development and progression of AD, with particular emphasis on the molecular pathways and key mediators involved in pathogen-triggered neuroinflammation.

## 1 Introduction

Alzheimer’s disease (AD) is the most common cause of dementia and is characterized by progressive neurodegeneration. It typically manifests with early episodic memory impairment, followed by deficits in language, executive function, and visuospatial abilities, ultimately leading to loss of independence and daily functioning. The neuropathological hallmarks of AD include extracellular amyloid-β (Aβ) plaques and intracellular neurofibrillary tangles (NFT) composed of hyperphosphorylated tau (p-tau), accompanied by synaptic and neuronal loss. In addition to these classical features, a substantial body of evidence has highlighted a prominent neuroinflammatory component in AD brains (Kiraly et al., 2023; Heneka et al., 2025). Neuroinflammation is generally defined as the immune response of the central nervous system (CNS) to harmful stimuli, such as toxic aggregates, infections, or traumatic injury. It is characterized by the production of inflammatory mediators, including cytokines, chemokines, nitric oxide, and reactive oxygen species (ROS) (Disabato et al., 2016). Chronic exposure to inflammatory signals can compromise the integrity of the blood–brain barrier (BBB) (Wu et al., 2026), facilitating the infiltration of peripheral immune cells and circulating cytokines into the CNS. Microglia and astrocytes, the principal resident immune cells of the brain, play a central role in AD-related neuroinflammation. Upon activation, they undergo morphological and functional changes and release pro-inflammatory mediators such as interleukin (IL)-1β and tumor necrosis factor α (TNF)-α. These factors can activate postsynaptic receptors and downstream signaling pathways, including nuclear factor κB (NF-κB), ultimately contributing to synaptic dysfunction, neuronal death, and impaired neurogenesis (Heneka et al., 2014; Micheau and Tschopp, 2003). Several hypotheses have been proposed to explain the origin of neuroinflammation in AD. One of the earliest suggests that Aβ oligomers and plaques, together with NFT, act as endogenous danger signals that activate microglia and astrocytes. These structures are recognized as “non-self” or damage-associated molecular patterns (DAMP), triggering a sustained immune response. Persistent activation leads to prolonged secretion of pro-inflammatory cytokines and neurotoxic factors, thereby promoting chronic neuroinflammation and neurodegeneration (Mcquade and Blurton-Jones, 2019; Onyango et al., 2021). An alternative hypothesis links AD onset and progression to “neuroinflammaging,” the brain-specific counterpart of the systemic, age-related inflammatory condition known as inflammaging (Franceschi et al., 2000). In this framework, neuroinflammation is considered an early and potentially primary event. Aging is associated with the upregulation of pro-inflammatory pathways in glial cells (Neal and Richardson, 2018; Walker et al., 2022; Muzio et al., 2021), a condition that has been shown to impair Aβ clearance, increase β-site APP cleaving enzyme 1 (*BACE1*) expression, and promote tau phosphorylation. These processes may, in turn, initiate and accelerate neurodegenerative proteinopathies (Soraci et al., 2024). In this review, we explore the hypothesis that infections may act as a trigger, or at least as an important contributor of neuroinflammation in AD, although most available evidence remains associative rather than causal. This concept is supported by multiple lines of evidence, including postmortem analyses of AD brain tissue, epidemiological studies, and the intrinsic antimicrobial properties of proteins involved in AD pathology, such as Aβ. For example, in mouse models of β-amyloidosis, experimental sepsis has been shown to enhance fibrillar amyloid deposition, amplify neuroinflammation, and worsen cognitive deficits (Basak et al., 2021). Moreover, numerous studies indicate that infectious agents may represent significant risk factors for CNS inflammation (Auvin et al., 2010; Sampson et al., 2016; Tran et al., 2021). When pathogens or their components reach the CNS, they can activate innate immune responses, leading to neuroinflammation. This response may result in neuronal damage and death or exacerbate pre-existing inflammatory states. However, the mechanisms linking peripheral infections to central neurodegenerative processes remain only partially understood, and likely involve a complex interplay between age-related immune dysfunction, BBB impairment secondary to AD pathology, and opportunistic colonization. Indeed, pathogen-driven contribution to neuroinflammation may also stem from the depletion of naïve T and B lymphocytes, reduced phagocytic capacity, degradation of BBB physical integrity, or a weakened immune system coupled with the disruption of the normal microbiota, thereby allowing pathogens to exert their detrimental effects on the AD brains. Here, we review current evidence on the contribution of various pathogens and related factors to AD-associated neuroinflammation, including viral, bacterial and fungal infections. In addition, we will explore the connection between gut microbiota and the brain, and the role of BBB in controlling neuroinflammation.

## 2 Astrocyte and microglia role in AD neuroinflammation

Multiple lines of evidence from epidemiological, neuropathological, and genetic studies support a central pathological role of neuroinflammation in AD. While the initial inflammatory response may exert protective effects, its chronic and dysregulated activation contributes to disease progression (Adamu et al., 2024). Microglia and astrocytes, the principal immune-competent cells of the CNS, are the main mediators of this neuroinflammatory response (Kwon and Koh, 2020). Microglia are macrophage-like glial cells that perform essential functions under both physiological and pathological conditions. They play a critical role in synaptic remodeling and structural plasticity and secrete growth factors that support neuronal survival. In response to injury or infection, microglia become activated, releasing cytokines and other inflammatory mediators while phagocytosing cellular debris and foreign particles. Transcriptomic analyses of microglia isolated from cognitively healthy individuals have identified a characteristic homeostatic gene expression profile (Galatro et al., 2017; Gosselin et al., 2017), including microglia-enriched surface receptor genes such as C-X3-C motif chemokine receptor 1 (*CX3CR1*), purinergic P2Y12-receptor (*P2RY12*), and transmembrane protein 119 (*TMEM119*). Overall, human microglia core signature comprises the expression of genes associated with immune response, cytokine production, and cell migration, reflecting the characteristic features of the homeostatic state. In contrast, microglia from AD brains exhibit distinct activation states associated with disease progression (Olah et al., 2020). Specifically, disease-associated microglia (DAM) are characterized by downregulation of homeostatic genes and upregulation of genes involved in endocytosis/phagocytosis (cluster of differentiation 68 (*CD68*), cathepsin D (*CTSD*), cystatin F (*CST7*)), lipid metabolism (apolipoprotein E (*APOE*), lipoprotein lipase (*LPL*)), immune signaling (triggering receptor expressed on myeloid cells 2 (*TREM2*), TYRO protein tyrosine kinase binding protein (*TYROBP*), integrin subunit alpha X (*ITGAX*)) and inflammatory response (secreted phosphoprotein 1 (*SPP1*), C-C motif chemokine ligand 2 (*CCL2*)). In addition, microgliosis driven by increased proliferation and activation of microglia represents a key feature of AD and has been shown to predict the onset of cognitive decline (Malpetti et al., 2020). This process has been associated with activation of the colony-stimulating factor 1 receptor (CSF1R) signaling pathway (Gomez-Nicola et al., 2013). Astrocytes, the other major glial population involved in AD-related neuroinflammation, play essential roles in maintaining CNS homeostasis. These include regulation of the BBB, modulation of synaptic activity through neurotransmitter uptake and release, and clearance of neurotoxic molecules (Verkhratsky and Nedergaard, 2018). In response to pathological stimuli, astrocytes undergo reactive gliosis, characterized by morphological and molecular changes that contribute to neuroinflammation. In AD, reactive astrocytes display hypertrophy of cellular processes, increased expression of cytoskeletal proteins such as glial fibrillary acidic protein (GFAP) and vimentin, and upregulation of genes associated with innate immunity, including lipocalin-2 (*LCN2*) and α1-antichymotrypsin (*SERPINA3*) (Zhou et al., 2020). Notably, these alterations often precede the onset of clinical symptoms, suggesting that astrocyte dysfunction may represent an early event in AD pathogenesis (Shah et al., 2022). Reactive astrocytes are frequently localized near Aβ plaques, where they contribute to the degradation and clearance of abnormal protein aggregates (Verkhratsky et al., 2023). Astrocyte reactivity is also associated with functional changes, including excessive release of γ-aminobutyric acid (GABA) and glutamate, which can lead to synaptic dysfunction (Liddelow et al., 2017). The shift from homeostatic support to immune activation and aggregate clearance is therefore accompanied by increased neuronal and synaptic damage (Verkhratsky et al., 2019). Given that a single astrocyte can interact with up to two million synapses in the human brain, even subtle alterations in astrocyte function may have profound consequences on neuronal network activity and cognitive performance. Overall, the interplay between microglial activation, astrocyte reactivity, and sustained inflammatory signaling creates a self-perpetuating cycle that exacerbates neuronal damage and accelerates neurodegeneration (**Figure 1**).

**Figure 1:**
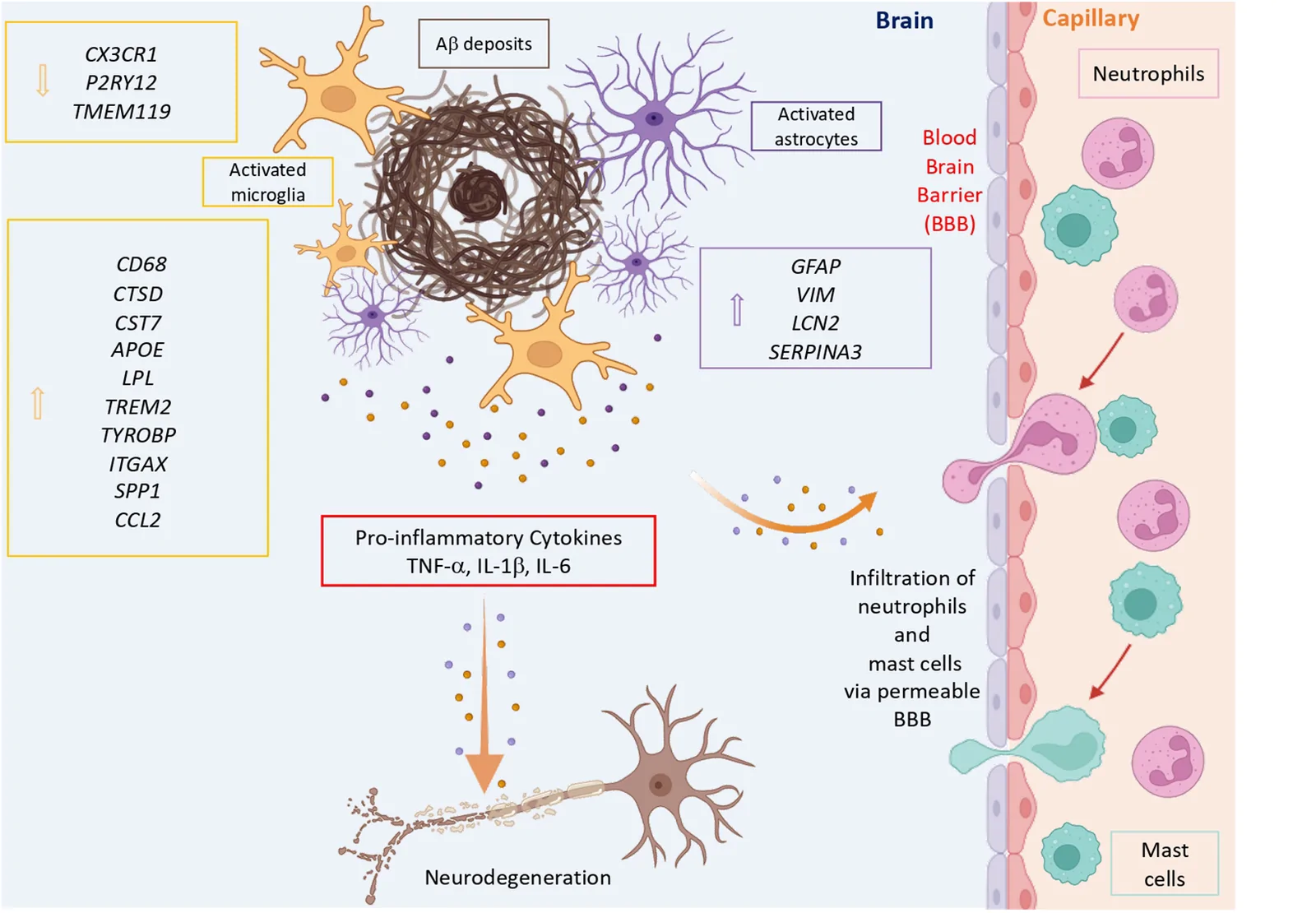
Neuroinflammatory mechanisms involved in Alzheimer’s disease. Amyloid-β (Aβ) deposition promotes the activation of microglia and astrocytes, leading to the release of pro-inflammatory cytokines, including TNF-α, IL-1β, and IL-6. Activated microglia exhibit downregulation of homeostatic genes such as *CX3CR1, P2RY12, TMEM119*, and upregulation of disease-associated microglia genes including *CD68, CTSD, CST7, APOE, LPL, TREM2, TYROBP, ITGAX, SPP1*, and *CCL2*. Reactive astrocytes display increased expression of *GFAP, VIM, LCN2*, and *SERPINA3*. Chronic neuroinflammation contributes to blood–brain barrier (BBB) dysfunction, facilitating infiltration of peripheral immune cells, including neutrophils and mast cells, ultimately promoting neuronal damage and neurodegeneration. TNF-α = tumor necrosis factor-α; IL-1β = interleukin-1β; IL-6 = interleukin-6; *CX3CR1* = C-X3-C motif chemokine receptor 1; *P2RY12* = purinergic P2Y12-receptor; *TMEM119* = transmembrane protein 119; *CD68* = cluster of differentiation 68; *CTSD* = cathepsin D; *CST7* = cystatin F; *APOE* = apolipoprotein E; *LPL* = lipoprotein lipase; *TREM2* = triggering receptor expressed on myeloid cells 2; *TYROBP* = TYRO protein tyrosine kinase binding protein; *ITGAX* = integrin subunit alpha X; *SPP1* = secreted phosphoprotein 1; *CCL2* = C-C motif chemokine ligand 2; *GFAP* = glial fibrillary acidic protein; *VIM* = vimentin; *LCN2* = lipocalin-2; SERPINA3 = serine protease inhibitor clade A member 3.

## 3 Infectious pathogens associated with AD

AD diagnosis is often preceded by early manifestations such as cognitive decline, behavioral changes, and sensory dysfunction (Bathini et al., 2024). Although the exact mechanisms linking these symptoms to severe dementia are not fully understood, growing evidence supports the involvement of diverse pathogens, both neurotropic and non-neurotropic, in AD. This has led to the formulation of a “pathogen hypothesis” of AD, particularly relevant in the older population (Harris and Harris, 2015). Pathogens encompass virtually all major microbial groups, including bacteria, fungi, viruses, and protozoa, and are increasingly recognized as potential contributors to the neuronal inflammation underlying disease onset and progression (Shin et al., 2024) (**Figure 2**). However, to reliably evaluate the association of such microorganisms with AD, consensus protocols and concerted efforts are needed, focusing the attention on the identification of AD pathobiome (Lathe et al., 2023). The main pathogens associated with AD to date include human herpesviruses, influenza viruses, severe acute respiratory syndrome coronavirus 2 (SARS-CoV-2), several bacterial species including *Chlamydia pneumoniae, Porphyromonas gingivalis*, and *Helicobacter pylori*, and various fungal species. Importantly, these pathogens may contribute to AD pathogenesis through both direct neuroinvasion and indirect systemic inflammatory responses, highlighting the multifactorial nature of infection-driven neurodegeneration.

**Figure 2:**
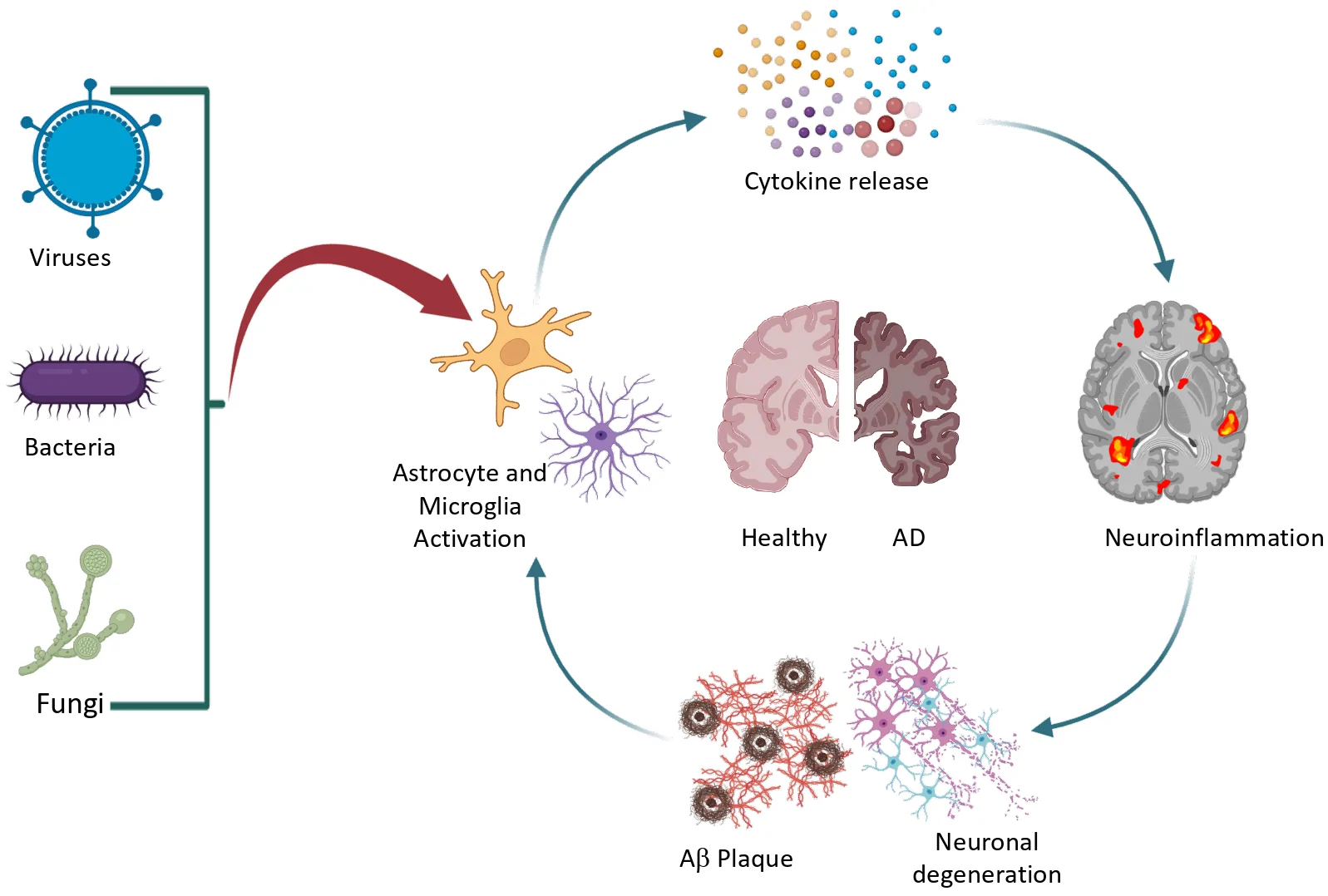
Overview of the contribution of infectious agents to Alzheimer’s disease (AD) pathogenesis. Viruses, bacteria, and fungi can activate astrocytes and microglia, triggering the release of inflammatory cytokines and promoting a chronic neuroinflammatory environment. Sustained inflammation contributes to Aβ plaque deposition, neuronal degeneration, and progressive transition from a healthy brain state to AD-associated neuropathology. The figure highlights the self-amplifying cycle linking pathogen-induced immune activation, neuroinflammation, and neurodegeneration.

### 3.1 Viral pathogens

Viral infections, mainly those sustained by Herpes viruses, have been long associated with the insurgence of AD. Their contribution to the pathogen hypothesis is supported by a great body of literature, detailing viral detection, pathogenic mechanisms and vaccination in the context of dementia onset and progression.

#### 3.1.1 Human herpesviruses

Herpesviruses are characterized by a double-stranded DNA genome and a complex structure consisting of four distinct components: a core containing the genome, an icosahedral capsid, an amorphous proteinaceous tegument, and a lipid envelope. Although more than 100 herpesvirus species have been identified, only eight are known human pathogens: herpes simplex virus type 1 and 2 (HSV-1 and HSV-2), varicella-zoster virus (VZV), Epstein–Barr virus (EBV), cytomegalovirus (CMV), and human herpesviruses 6, 7, and 8 (HHV-6, HHV-7 and HHV-8) (Whitley, 1996). Human herpesviruses, particularly HSV-1 and HSV-2, are known to establish lifelong latency and to exhibit periodic reactivation events (Cunningham et al., 2006) often resulting in a persistent inflammatory state, consistent with the neuroinflammation observed in AD (Licastro and Porcellini, 2016). Among the various herpesviruses, HSV-1 is the most strongly implicated in the development and progression of AD. Therefore, our analysis of herpesvirus-driven neuroinflammation in AD will mainly focus on this species. In older individuals, age-related immune decline may facilitate HSV-1 migration to the temporal cortex, potentially causing encephalitis, inflammation, and the development of AD hallmarks such as Aβ plaque deposition (Itzhaki, 2018). Notably, Aβ peptides exhibit antimicrobial properties, acting against microbial membranes (Sood et al., 2008), bacterial lipopolysaccharides (LPS) (Wang et al., 2014), and by sequestering pathogens through fibril formation (Torrent et al., 2012). In the context of HSV-1 infection, Aβ aggregation appears to play a protective role by preventing viral fusion with host cell membranes (Bourgade et al., 2016). In murine models, Aβ fibril formation significantly reduced viral lethality, even at nanomolar concentrations (Eimer et al., 2018). This mechanism may explain the frequent co-localization of HSV-1 and Aβ plaques in the human brain. Indeed, Wozniak et al. demonstrated this association in temporal and frontal cortices using *in situ* polymerase chain reaction (PCR) and thioflavin S staining, with significantly higher prevalence in AD patients compared to age-matched controls (Wozniak et al., 2009). An emerging area of interest is the role of viral microRNA (miRNA), which have been demonstrated to regulate the expression of host genes involved in Aβ degradation, oligodendrocyte development, myelination, and synaptic vesicle recycling (Hemmat et al., 2022). HSV infection has also been shown to induce tau hyperphosphorylation, promoting aggregation and prion-like propagation (Powell-Doherty et al., 2020; Ijezie et al., 2025). Epidemiological studies further support the link between HSV-1 and AD. Longitudinal prospective population studies evaluated anti-HSV antibodies in blood serum and found that high levels of anti-HSV IgM antibodies (a marker of active viral reactivation) were associated with a significantly higher risk of developing AD over a >10-year follow-up period (Letenneur et al., 2008; Lovheim et al., 2015). A large-scale, retrospective matched case-control study, using the IQVIA PharMetrics database of over 344,000 AD case-control pairs aged ≥ 50, demonstrated that a recorded history of HSV-1 infection was significantly associated with an increased likelihood of AD (adjusted OR: 1.80; 95% CI: 1.65–1.96) (Liu et al., 2025). Viral DNA has been frequently detected in postmortem AD brains, and the presence of the ApoE ϵ4 allele has been associated with increased susceptibility to both HSV infection and AD (Linard et al., 2020). Similar associations have been reported for HSV-2 and VZV (Biagio et al., 2024). Large-scale longitudinal evidence indicates that recurrent VZV infection is associated with a higher risk of dementia compared to a single VZV episode (Polisky et al., 2025). In particular, co-infections appear to exacerbate disease progression, suggesting synergistic pathogenic interactions among herpesviruses. However, there are some studies reporting negative or null findings alongside epidemiological criticisms regarding direct link between HSV-1 infection or brain viral load and AD. In a post-mortem brain tissue study using PCR analysis, researchers found HSV-1 DNA in 74% of AD brains and 73% of control brains, demonstrating no statistically significant difference in viral presence between cases and controls. Furthermore, they found no significant interaction with the ApoE ϵ4 allele (Beffert et al., 1998). In another population-based study following 1,915 non-demented participants over an average of 9.1 years, HSV-1 seropositivity was associated with the minor cognitive domain decline, but failed to show an increased risk for incident dementia or AD (adjusted hazard ratio = 1.13, 95% CI: 0.77–1.66) (Murphy et al., 2021). Another important criticism to the HSV-1 hypothesis is the disparity between global infection rates and AD incidence, with HSV-1 seroprevalence accounting for 60% to 80% of adults globally (and >80% in adults aged 65+) while AD prevalence is estimated about 10% to 15% of adults aged 65 +. To address this discrepancy, it has been argued that HSV-1 may act only as a conditional risk factor under specific circumstances such as presence of ApoE ϵ4 allele, actual entry of the virus into the CNS, and co-presence of other risk factors like age-related immunosenescence, vascular risk factors, and compromised BBB integrity (Tyler, 2021; Mangold and Szpara, 2019). Importantly, anti-herpetic treatments provide indirect support for a causal relationship. Acyclovir has been shown to reduce Aβ deposition and tau phosphorylation (Harris and Harris, 2018), although it lacks anti-inflammatory effects. Vaccination and strong humoral responses against herpesviruses have also been associated with reduced AD incidence (Agostini et al., 2016; Lehrer and Rheinstein, 2021).

#### 3.1.2 Influenza viruses

Influenza viruses belong to the Orthomyxoviridae family and are among the most widespread viral pathogens worldwide (Voskarides et al., 2018). These are enveloped viruses, with a lipid membrane derived from the host cell that contains two key glycoproteins essential for viral infectivity: hemagglutinin (HA), which mediates binding to host cell receptors and membrane fusion (De Bruin et al., 2022), and neuraminidase, which facilitates viral release from infected cells (Kim et al., 2019). Variations in these glycoproteins define the different viral subtypes. Influenza viruses primarily cause acute respiratory infections characterized by symptoms such as fever, malaise, myalgia, headache, sore throat, cough, and fatigue (Falsey et al., 2022). Although influenza viruses predominantly infect the respiratory tract, accumulating evidence indicates their ability to invade and persist in the CNS (Van Riel et al., 2015). In a murine model, H5N1 virus was initially detected in the enteric nervous system and subsequently spread through the peripheral nervous system to the CNS within three days post-infection (Jang et al., 2009). Once in the CNS, influenza viruses can infect neurons and microglial cells, inducing pathological processes such as increased phosphorylation of α-synuclein, a protein implicated in neurodegenerative diseases including Parkinson’s disease (PD) and AD (Twohig and Nielsen, 2019). Notably, even after viral clearance (typically within 21 days post-infection), a persistent immune response characterized by microgliosis has been observed. This sustained neuroinflammatory state may contribute to neurodegenerative processes consistent with AD pathology. Similarly, the H1N1 influenza strain has been shown to induce robust production of pro-inflammatory cytokines, including IL-1β, IL-6, TNF-α, and monocyte chemoattractant protein, thereby promoting neuroinflammation (Ding et al., 2022). Although viral replication in brain cells varies depending on specific mutations in HA (Sun et al., 2010), the associated cytokine storm can disrupt BBB integrity, alter glutamatergic synaptic transmission, and induce progressive neuronal dysfunction (Dusedau et al., 2021). Hosseini et al. demonstrated that even non-neurotropic influenza strains can indirectly affect the CNS (Hosseini et al., 2021), activating a peripheral immune response and causing reduced dendritic spine density, impaired synaptic connectivity, and cognitive deficits, further supporting a link between influenza infection and AD-related neuropathology. However, most experimental evidence derives from murine models of infections, while reports of a direct association between the influenza virus and AD in humans remain limited. An indirect proof of the link between the two pathologies is provided by epidemiological studies regarding the efficacy of influenza vaccination. A large retrospective study conducted in individuals aged 65 years and older reported that influenza vaccination was associated with a reduced risk of developing AD, with approximately 5% lower incidence over a four-year follow-up period (Bukhbinder et al., 2022). A subsequent study indicated that high-dose influenza vaccines may further enhance this protective effect, particularly in female subjects (Bukhbinder et al., 2026). However, limitations such as short follow-up duration and lack of detailed demographic and clinical data preclude definitive conclusions. The mechanisms underlying the protective effect of influenza vaccination in AD remain to be fully elucidated. It has been proposed that immune stimulation induced by vaccination may enhance clearance of Aβ plaques and mitigate chronic neuroinflammation. Yang et al. demonstrated in APP/PS1 mice that influenza vaccination reduced Aβ plaque burden in both the cortex and hippocampus and enhanced microglial activation, particularly in terms of Aβ phagocytosis (Yang et al., 2020). In addition, vaccination modulated cytokine production, promoting a more balanced inflammatory response and contributing to brain homeostasis. Cognitive deficits in transgenic mice were also significantly improved following vaccination. Influenza-based vaccination strategies may also provide a novel therapeutic approach for AD. Chimeric influenza vaccines expressing Aβ epitopes have shown promising results. Davtyan and colleagues developed an inactivated influenza virus vaccine carrying a B-cell epitope of Aβ, which not only protected against viral infection but also induced antibodies capable of binding Aβ in its monomeric, oligomeric, and fibrillar forms, reducing its deposition and associated toxicity (Davtyan et al., 2011). These findings suggest that influenza-based immunization strategies may represent a dual therapeutic approach, targeting both infectious agents and neurodegenerative mechanisms.

#### 3.1.3 SARS-CoV-2

SARS-CoV-2, the causative agent of the COVID-19 pandemic, is an enveloped positive-sense single-stranded RNA virus belonging to the β-coronavirus family. Since its emergence in late 2019, SARS-CoV-2 has caused more than 700 million infections and over 7 million deaths worldwide. This virus is primarily transmitted through respiratory droplets and contact with infected individuals or contaminated surfaces (Yu et al., 2004; Otter et al., 2016). Clinical manifestations range from mild flu-like symptoms, including fever, cough, fatigue, myalgia, and gastrointestinal (GI) disturbances, to severe pneumonia associated with respiratory and renal failure (Wang et al., 2020). A hallmark of severe COVID-19 is the development of a “cytokine storm,” characterized by excessive production of inflammatory mediators that can lead to disseminated intravascular coagulation, vascular leakage, and systemic endothelial dysfunction (Moore and June, 2020; Tang et al., 2020). In addition, many patients experience persistent post-acute sequelae, collectively referred to as “long COVID,” which is now recognized as a chronic condition associated with prolonged neurological and systemic symptoms including memory impairment, “brain fog,” motor dysfunction, and delirium, resembling several features of dementia (Peluso and Deeks, 2024). Long-term cognitive decline has also been documented in critically ill patients following infection (Hosseininasab et al., 2024). Neuroimaging studies have revealed persistent structural brain alterations after COVID-19 resolution, including gray matter atrophy, white matter abnormalities, and reduced cerebral vascularization, even in individuals without overt neurological symptoms (Qin et al., 2021). Increasing evidence indicates that SARS-CoV-2 can affect the CNS, as demonstrated by the detection of viral RNA and antigens in postmortem brain samples (Matschke et al., 2020). Several mechanisms have been proposed to explain CNS invasion, including direct infection of endothelial cells and disruption of the BBB, axonal transport through the olfactory nerve and dorsal root ganglia, and transmission via the vagus nerve and enteric nervous system (Dong et al., 2020; Mao et al., 2020; Shiers et al., 2020; Esposito et al., 2020). The intense inflammatory response observed in COVID-19 patients and the recovery of viral RNA and antigens in brain tissues have raised increasing interest regarding SARS-CoV-2 potential contribution to CNS impairment. Proteomic analyses in these patients have revealed alterations in proteins involved in neuronal metabolism, energy production, and cell death, such as increased expression of α-synuclein and caspase-3 (Mahin et al., 2024). Importantly, α-synuclein can interact with the virus spike protein, Aβ, and tau, while caspase-3 is a well-known mediator of neuronal apoptosis and AD-associated neurodegeneration (Idrees and Kumar, 2021; Louneva et al., 2008). Several neuropathological similarities between AD and COVID-19 have been proposed. Both conditions are characterized by astrocytosis, oxidative stress, and olfactory dysfunction. Furthermore, SARS-CoV-2 infection may alter intracellular Ca^2+^ homeostasis, promoting tau pathology and neuronal dysfunction (Reiken et al., 2022). Some epidemiological studies suggest that COVID-19 infection is associated with an increased risk of AD development, particularly in elderly individuals and women (Wang et al., 2022). Conversely, individuals with AD exhibit an increased susceptibility to severe COVID-19 outcomes, a risk that is partly attributable to the *APOE ϵ4* genotype. Notably, APOE is expressed not only in astrocytes but also in macrophages and lung epithelial cells, suggesting a potential role in mediating the heightened severity of COVID-19 observed in ϵ4 carriers (Xiong et al., 2021). This evidence has led to the hypothesis that severe or prolonged neuroinflammation after COVID-19 might accelerate existing neurodegenerative processes in susceptible individuals. However, COVID-19 related cognitive symptoms are a distinct syndrome that shares some mechanistic features with AD but should not currently be considered equivalent to Alzheimer’s pathology. There is ongoing research into whether COVID-19 may accelerate or increase the risk of neurodegeneration in some susceptible individuals, but the evidence is not yet conclusive.

### 3.2 Bacterial pathogens

Alongside viruses, bacterial pathogens have been extensively associated with AD pathogenesis through both direct CNS infection and the induction of chronic inflammatory responses. Bacteria can contribute to neurodegeneration through multiple mechanisms, including gut dysbiosis, disruption of endothelial barriers, transcytosis across the BBB, and migration through cranial nerves. Gut dysbiosis is increasingly recognized as a major contributor to AD development (Jia et al., 2025). Alterations in gut microbiota composition may compromise intestinal barrier integrity, allowing pathogenic bacteria and inflammatory mediators to enter the bloodstream and eventually reach the brain (Alam et al., 2021). In parallel, systemic inflammation triggered by dysbiosis may promote Aβ deposition and neuroinflammation (Ullah et al., 2025). The complex interplay between gut dysbiosis and brain alterations will be discussed in detail in Chapter 4. Bacterial toxins further contribute to CNS damage by altering endothelial permeability and directly affecting neuronal survival. In particular, LPS, a major endotoxin produced by Gram-negative bacteria, has been detected at significantly higher levels in the plasma of AD patients compared to healthy controls (Brown, 2019). LPS and other bacterial toxins can disrupt BBB integrity, activate microglia, and induce neuronal injury (Fettucciari et al., 2017). Bacteria may also bypass endothelial barriers through transcytosis, a vesicle-mediated transport mechanism that allows intracellular passage across endothelial cells (Gradstedt et al., 2013). In addition, several pathogens can invade the CNS through cranial nerves, particularly the olfactory and trigeminal nerves. This mechanism is particularly relevant considering that olfactory dysfunction often precedes AD diagnosis (Meyer et al., 2025).

#### 3.2.1 Porphyromonas gingivalis

Oral pathogens have attracted major interest as potential contributors to neurodegeneration. Among them, *Porphyromonas gingivalis*, a Gram-negative anaerobic bacterium responsible for chronic periodontitis, represents one of the most extensively investigated bacterial contributors to AD. Translocation of *P. gingivalis* or its toxic virulence factors from the oral cavity into the central nervous system—facilitated by systemic circulation or peripheral nerve pathways—disrupts blood-brain barrier integrity and leads to central brain colonization. Within the brain parenchyma, bacterial components, particularly LPS and toxic cysteine proteases known as gingipains, act as potent inflammatory triggers capable of degrading antimicrobial and anti-inflammatory proteins, modulating macrophage activity, and promoting inflammatory cytokine release (Olsen, 2021). Consistently, recent *in vivo* evidence in zebrafish larvae showed that *P. gingivalis* disrupts BBB integrity in a gingipain-dependent manner through degradation of tight junction proteins in cerebral vessels (Mieszkowska et al., 2025).These virulence factors persistently activate microglial cells and astrocytes via pattern recognition receptors, such as Toll-like receptor 4 (TLR4), triggering downstream NF-κB signaling and the sustained release of pro-inflammatory cytokines alongside ROS. This chronic, bacterial-driven neuroinflammation directly exacerbates classic AD neuropathological hallmarks by Aβ aggregation and tau hyperphosphorylation, establishing a self-sustaining neurodestructive cycle that drives synaptic loss and neurodegeneration. The pathogen has been detected directly in the brains of AD patients, supporting a possible contribution to disease pathogenesis. Moreover, elevated serum immunoglobulins against *P. gingivalis* have been associated with increased mortality risk in AD patients older than 65 years (Beydoun et al., 2020).

#### 3.2.2 Spirochetes

Spirochetes have also been implicated in AD pathogenesis. These neurotropic bacteria, including *Treponema pallidum* and *Borrelia burgdorferi*, are known to cause meningitis, encephalitis, and Lyme disease (Wen et al., 2022). Neurosyphilis, the late neurological manifestation of *T. pallidum* infection, is associated with chronic neuroinflammation, synaptic dysfunction, tau pathology, neuronal loss, and cognitive decline (Fadel et al., 2024). Once within the CNS, spirochetes may form biofilms that protect them from immune clearance. These biofilms activate Toll-like receptor 2 (TLR2), leading to TNF-α and NF-κB activation, thereby sustaining neuroinflammation and amyloid deposition (Allen, 2016). Notably, several spirochetes produce bacterial amyloids that may directly interact with Aβ aggregates and amplify inflammatory responses.

#### 3.2.3 Chlamydia pneumoniae

Another pathogen strongly associated with AD is *Chlamydia pneumoniae*, an intracellular bacterium frequently detected in AD brains, particularly in late-onset forms of the disease (Gerard et al., 2006; Balin et al., 2008). *C. pneumoniae* can infect neurons, astrocytes, and glial cells and has been identified in close proximity to Aβ plaques. The pathogen may access the CNS through a compromised BBB or through infected immune cells acting as Trojan horses (Kortesoja et al., 2020). Moreover, *C. pneumoniae* was shown to infect the olfactory and trigeminal nerves in a murine infection model and to propagate to CNS within 72h post-infection modulating the host gene expression and favoring the AD risk (Chacko et al., 2022). Age-related macular degeneration (AMD) is a retinal disease that shares neuroinflammatory pathways with AD (Butovsky and Rosenzweig, 2025) and in which retinal parainflammation plays a major role, with a potential involvement of various infectious agents (Xu et al., 2009; Larsen et al., 2025). Different studies have established a significant serological association between AMD and *C. pneumoniae* infection (Shen et al., 2009; Nakata et al., 2015) and between antibody titers of *C. pneumoniae* and risk of AMD progression (Robman et al., 2005). Of note, *C. pneumoniae* inclusions were recently identified in the postmortem retina (Gaire et al., 2026), showing higher burden in AD retinal and brain tissues than controls, increasing with APOE ϵ4 positivity, disease stage, and cognitive impairment. Persistent intracellular infection and activation of inflammatory pathways, including the NLRP3 inflammasome, may contribute to the progression of AD and motivate NLRP3-targeted and/or antibiotic-based early treatment strategies (Gaire et al., 2026).

#### 3.2.4 Pseudomonas aeruginosa

*Pseudomonas aeruginosa*, an opportunistic Gram-negative pathogen, has increasingly been recognized as a potential contributor to the etiology of AD and its associated neuroinflammatory cascades (Zhan et al., 2016). Peripheral or systemic infections by *P. aeruginosa* can compromise the structural integrity of the BBB through the release of potent virulence factors, such as pyocyanin, elastase, and LPS, thereby facilitating pathogen transit or the entry of pro-inflammatory bacterial metabolites into the CNS. Upon reaching the brain parenchyma, *P. aeruginosa* LPS persistently activates microglial cells and reactive astrocytes via TLR4 and NF-κB signaling pathways, driving the sustained secretion of pro-inflammatory cytokines and ROS. Furthermore, *P. aeruginosa* secretes functional bacterial amyloid proteins (such as FapC) to form resilient biofilms; these bacterial amyloids can cross-seed with human Aβ, accelerating host protein misfolding and aggregation (Javed et al., 2020). Cross-sectional microbiome profiling in human clinical cohorts reveals an altered abundance of the *Pseudomonas* genus in both the oral and gut dysbiotic signatures of AD patients compared to cognitively healthy controls, where specific microbial taxa correlate with decreased Mini-Mental State Examination (MMSE) scores (Xi et al., 2021). Furthermore, post-mortem brain tissue analyses have demonstrated a significantly higher burden of Gram-negative bacterial components in the parenchyma and cerebral vasculature of AD brains relative to age-matched controls (Zhan et al., 2016). This clinical evidence supports the epidemiological hypothesis that systemic or localized *P. aeruginosa* burden promotes persistent peripheral-to-central inflammatory signaling, breaching the blood-brain barrier and accelerating the clinical onset and trajectory of AD.

#### 3.2.5 Helicobacter pylori

Bacterial extracellular vesicles may represent an additional indirect mechanism linking infection to AD. Outer membrane vesicles released by *Helicobacter pylori* have been shown to disseminate systemically and reach the brain, where they promote Aβ deposition, microglial dysfunction, complement activation, and cognitive impairment in experimental models (Xie et al., 2023). *H. pylori* has been investigated as a potential contributor to AD pathogenesis, although findings remain inconsistent. Some studies suggest that infection may exacerbate gut-brain axis dysfunction, promote BBB disruption, and enhance neuroinflammatory processes. In a clinical study, infected AD patients showed worse cognitive performance together with increased CSF levels of BBB-related markers, phosphorylated tau, and altered gut microbiota and metabolites (Li et al., 2026b). A recent systematic review and meta-analysis reported an increased risk of AD associated with *H. pylori* infection, but with substantial heterogeneity (Du et al., 2025). Importantly, population-based data did not support a clear protective effect of eradication therapy on AD risk (Keranen et al., 2025). Overall, *H. pylori* may act as a modulatory factor within the gut–brain axis rather than a direct causal driver of AD. Interestingly, not all bacteria exert detrimental effects. Modulation of gut microbiota through probiotic administration has shown beneficial effects in experimental AD models. Multi-strain probiotics were reported to improve cognitive performance, reduce Aβ deposition and tau phosphorylation, enhance synaptic plasticity, and attenuate microglial and astrocytic activation through modulation of inflammatory pathways, including AKT/GSK-3β signaling, in SAMP8 mice (Xiao-Hang et al., 2024). However, current evidence regarding the use of probiotics for individuals with dementia is insufficient to support their clinical application (Kruger et al., 2021).

### 3.3 Fungal infections

Although less extensively investigated than viral and bacterial pathogens, fungi are increasingly recognized as potential contributors to AD pathogenesis. Fungi represent a highly heterogeneous group of eukaryotic microorganisms widely distributed in the environment and characterized by remarkable adaptability and invasive potential. While only a limited subset of fungal species acts as human pathogens, fungal infections can become persistent and invasive, affecting the skin, respiratory system, GI tract, and CNS, particularly in immunocompromised individuals (Phuna and Madhavan, 2022). Fungi are generally classified as yeasts, filamentous fungi (molds), or dimorphic fungi. Importantly, the ability to switch between yeast-like and filamentous morphologies represents a major virulence factor that facilitates tissue invasion and host colonization (Maresca and Kobayashi, 1989). Although fungal infections are most commonly associated with skin and soft tissue diseases, invasive pulmonary, bloodstream, urinary tract, and CNS infections are well documented, particularly in individuals with chronic diseases or immunosuppression (Mohamed et al., 2023). The association between fungal infections and AD emerged from clinical observations in which cryptococcal meningitis was initially misdiagnosed as dementia due to overlapping neurological manifestations, including memory impairment, behavioral alterations, motor dysfunction, and cognitive decline (Ala et al., 2004). Remarkably, these symptoms improved following antifungal treatment, suggesting an infectious rather than a neurodegenerative disease (Rafael, 2005). These observations provided the first evidence supporting a possible relationship between invasive fungal infections, chronic neuroinflammation, and AD-like pathology. Subsequent studies identified fungal components directly in the serum and brain tissue of AD patients. Using histological, molecular, and proteomic approaches, Alonso and colleagues demonstrated the presence of fungal proteins, DNA, and polysaccharides, including (1–3)-β-glucans, at significantly higher levels in AD patients compared to healthy controls (Alonso et al., 2014a; 2014b). Interestingly, different fungal species were frequently detected simultaneously within the same patient, supporting the hypothesis that polymicrobial fungal infections may contribute to chronic neuroinflammation and patient-specific disease progression. Further evidence demonstrated that fungi may adopt intracellular localization within the CNS, leading to the so-called “endomycoses” (Pisa et al., 2015a). Intracellular fungal elements were detected within neurons, endothelial cells, and neurovascular structures, often surrounding the nucleus. This intracellular localization likely provides protection from immune clearance while allowing persistent inflammatory activation and nutrient acquisition. Endothelial cells of the BBB can internalize fungal cells, particularly *Candida* spp., through pseudopod-mediated endocytosis, facilitating fungal dissemination into the CNS (Parady, 2018; Jong et al., 2001). Notably, the antimicrobial properties of Aβ peptides were demonstrated even against *Candida albicans* (Soscia et al., 2010). The spatial distribution of fungal species within AD brains further supports their pathological relevance. Fungal elements have been detected in the frontal cortex, cerebellum, hippocampus, entorhinal cortex, and choroid plexus (Pisa et al., 2015b). Remarkably, different fungal species were often identified in distinct brain regions, suggesting heterogeneous and patient-specific infection patterns rather than uniform colonization. This observation raises the possibility that fungal species may establish synergistic interactions that facilitate CNS persistence and chronic inflammation. Among the fungal genera most frequently detected in AD samples are *Candida*, *Alternaria*, *Cladosporium*, and *Malassezia* (Phuna and Madhavan, 2022). *Candida* spp. are thought to originate from intestinal fungal dysbiosis, which appears significantly increased in AD patients and may promote gut inflammation and fungal dissemination into the bloodstream (Ling et al., 2020). Their ability to adhere to and damage the BBB likely represents a major route of CNS invasion. Similarly, *Malassezia* spp. may disseminate through impaired skin barriers and have been associated with psoriasis, a chronic inflammatory condition proposed as a risk factor for AD (Kim et al., 2020). *Alternaria* spp., although primarily associated with allergic and respiratory diseases, can cause invasive infections in immunocompromised and transplanted patients and may reach the CNS following bloodstream dissemination (Salo et al., 2006; Cardona et al., 2020). *Cladosporium* spp., a widespread opportunistic fungus, has also been associated with brain abscesses characterized by severe inflammation, neuronal injury, memory impairment, and cognitive dysfunction (Ahmad et al., 2017). Interestingly, some fungal species may also exert protective effects against neurodegeneration. *Alternaria* spp. have been reported to produce acetylcholinesterase inhibitors, whereas *Cladosporium* spp. can produce huperzine A, a compound with potential neuroprotective activity (Dos Santos et al., 2018). Recent experimental evidence suggests that fungal–bacterial interactions may further enhance microbial pathogenicity and host immune activation. *In vitro* studies have shown that *Candida albicans* can facilitate *P. gingivalis* uptake by macrophages, thereby promoting bacterial dissemination, while co-exposure to both microorganisms induces a predominantly pro-inflammatory M1 macrophages polarization characterized by increased IL-6, IL-1β, and TNF-α expression, suggesting a synergistic mechanism through which fungal–bacterial interactions may amplify systemic inflammation and potentially contribute to neuroinflammatory processes relevant to AD (De Jongh et al., 2025). Overall, current evidence supports the hypothesis that fungal infections may contribute to AD through chronic neuroinflammation, BBB disruption, oxidative stress, and persistent CNS colonization. Indeed, a recent study evidenced a higher fungal presence in the oral microbiota of hospitalized AD patients in comparison to a matched non-AD control group, highlighting a significant association between the disease and hemolytic *Candida* spp (Golipoor et al., 2024). On the other hand, a randomized analysis of Health and Retirement Study (HRS) suggested that AD susceptibility is strongly influenced by the individual vulnerability rather than by the exposure to a specific pathogen (Ukraintseva et al., 2024). Thus, whether fungal detection reflects causative involvement, opportunistic colonization, or secondary infection remains incompletely understood, and further mechanistic and longitudinal studies are needed to clarify the exact contribution of fungi to neurodegenerative processes.

## 4 Microbiota-gut-brain-axis

The human microbiota comprises bacteria, yeasts, fungi, archaea, and viruses that colonize multiple anatomical sites, including the GI tract, skin, lungs, and oral cavity (Ursell et al., 2014). These microbial communities contribute to essential physiological processes such as nutrient absorption, vitamin synthesis, metabolic regulation, protection against pathogens, and modulation of innate and adaptive immune responses. Among these microbial ecosystems, the gut microbiota is considered one of the most relevant for maintaining systemic health (Shreiner et al., 2015). Under physiological conditions, the gut microbiota is characterized by stability, resilience, high taxonomic diversity, and a symbiotic relationship with the host. Although microbial composition varies across GI regions and is influenced by age, diet, lifestyle, and medication use, the gut microbiota is mainly dominated by Firmicutes, Bacteroidetes, Actinobacteria, Proteobacteria, Fusobacteria, and Verrucomicrobia, with Firmicutes and Bacteroidetes representing the predominant phyla (Laterza et al., 2016). In addition to bacteria, the gut ecosystem includes fungi such as *Candida*, *Saccharomyces*, *Malassezia*, and *Cladosporium*, as well as Archaea and viruses comprising bacteriophages, prophages, eukaryotic viruses, archaeal viruses, and recently identified virus-like entities such as obelisks (Auchtung et al., 2018; Zheludev et al., 2024). However, despite this diversity, most current knowledge of host–microbiota interactions remain focused on bacterial components. Dysbiosis, defined as an imbalance in microbial composition characterized by reduced diversity and expansion of pathogenic or opportunistic organisms, can impair immune homeostasis, disrupt intestinal barrier integrity, and promote disease development (Aguirre De Carcer, 2018). Through microbial metabolites, structural components, and signaling molecules, gut microorganisms exert effects that extend beyond the GI tract and influence systemic metabolism, inflammation, hematopoiesis, and immune regulation. Because of its extensive bidirectional communication with multiple organs through neural, endocrine, immune, and metabolic pathways, the gut microbiota has increasingly been regarded as a “vital organ” (Ahlawat et al., 2021). Large-scale initiatives, including the Human Microbiome Project, MetaHIT, the American Gut Project, and the British Gut Project, have greatly contributed to characterizing microbiota composition and function at the population level (Human Microbiome Jumpstart Reference Strains, C. et al., 2010; Qin et al., 2010; Mcdonald et al., 2018; Jackson et al., 2018). Among host–microbiota communication networks, the gut–brain axis represents one of the most extensively studied systems. This bidirectional axis allows the GI tract to influence brain function and enables central neural processes to modulate GI activity. Disruption of this reciprocal network has been associated with GI disorders, altered stress responses, behavioral changes, and cognitive impairment (Bernstein, 2017; Brenner et al., 2018; Dinan and Cryan, 2017; Foster et al., 2017; Arentsen et al., 2018). Consequently, the gut–brain axis has emerged as a promising therapeutic target for disorders affecting mental health and cognitive function (Clapp et al., 2017; Jiang et al., 2017). Several pathways mediate communication within the microbiota–gut–brain axis. Microbiota-derived neuromodulators, including serotonin (5-HT), GABA, tryptophan metabolites, indole, and 4-ethylphenylsulfate, can influence the autonomic nervous system, enteric nervous system (ENS), vagal afferents, and brainstem nuclei such as the nucleus tractus solitarius (Hsiao et al., 2013; Jaglin et al., 2018). Experimental studies have shown that microbial metabolites can modulate brain function and behavior; for instance, 4-ethylphenylsulfate administration induces anxiety-like phenotypes in mice (Lyte, 2014). As a major source of peripheral serotonin, the gut microbiota also activates ENS neurocircuitry through 5-HT receptors (De Vadder et al., 2018). The microbiota further interacts with the hypothalamic–pituitary–adrenal (HPA) axis, a central regulator of stress responses. HPA-derived mediators such as corticosterone can reshape microbial composition, while the microbiota modulates HPA activity. Germ-free mice exhibit HPA hyperactivity and elevated plasma corticosterone levels, whereas patients with irritable bowel syndrome and dysbiosis show exaggerated adrenocorticotropic hormone responses to corticotropin-releasing factor (Mayer, 2000; Sudo et al., 2004; Dinan et al., 2006). Although substantial progress has been made, the precise mechanisms governing microbiota–brain communication remain incompletely understood. Age-related changes in gut microbiota composition are increasingly recognized as contributors to “inflammaging” (Thevaranjan et al., 2018; Boren and Gershwin, 2004; Franceschi et al., 2001; Lencel and Magne, 2011). A key mechanism linking the gut microbiota to inflammaging and neurodegeneration involves microbiota-derived short-chain fatty acids (SCFA), including acetate, propionate, and butyrate, produced through dietary fiber fermentation. SCFA exert anti-inflammatory and immunomodulatory effects by inhibiting histone deacetylases (HDACs), suppressing NF-κB–mediated cytokine production, and promoting regulatory T-cell differentiation and function (Rooks and Garrett, 2016; Arpaia et al., 2013). Importantly, SCFA also influence microglia, the resident immune cells of the CNS, which are essential for neural homeostasis, synaptic plasticity, and neuroprotection (London et al., 2013; Tay et al., 2017). Disruption of SCFA signaling may impair microglial function and exacerbate neuroinflammation and neurodegeneration (Bayazid et al., 2021). These findings have stimulated the development of microbiome-targeted interventions, including prebiotics, probiotics, postbiotics, and fecal microbiota transplantation (FMT), aimed at restoring microbial balance, enhancing beneficial metabolite production, and re-establishing neuroimmune homeostasis (Boehme et al., 2020; Matt et al., 2018). Clinical trials further highlight the growing interest in precision medicine approaches based on microbiome modulation. For example, the Gut-PRO Study (2024–2026; NCT07413744) is evaluating the safety, feasibility, and efficacy of oral probiotic supplementation in individuals with AD, while AI-personalized microbiome intervention trials use individual microbial profiles to design dietary strategies targeting systemic and disease-related biomarkers. FMT from healthy donors is also emerging as a comprehensive strategy to restore microbial composition and function. Preclinical evidence indicates that FMT can increase microbial diversity, enhance SCFA production, suppress pro-inflammatory microglial activation, promote amyloid-β clearance, and improve cognitive performance in AD models (Grabrucker et al., 2023; Qin et al., 2025). Although less extensively characterized than the bacteriome, the gut virome is increasingly emerging as a relevant component of the microbiota–gut–brain axis in neurodegenerative disorders (Badillo-Pazmay et al., 2025). Recent metagenomic studies have reported reduced phage richness and compositional shifts in AD, including alterations in Lactococcus-associated phages, which may affect host–microbe metabolic balance through changes in lactic acid pathways relevant to neuronal energy metabolism and synaptic function (Ghorbani et al., 2023). Similar virome signatures have been observed in mild cognitive impairment, where specific bacteriophages show disease-associated enrichment and moderate diagnostic potential, with stronger associations in more severe cognitive decline (Chaudhari et al., 2023). Longitudinal and cross-sectional studies further indicate that age-related and disease-associated virome remodeling is accompanied by increased lysogenic phages, reduced viral diversity, and altered phage–bacteria–metabolism interactions, suggesting a potential role of the virome in shaping microbial ecology, metabolic output, and neuroinflammatory pathways during cognitive aging (James et al., 2024). Overall, the microbiota–gut–brain axis provides a mechanistic link between peripheral microbial imbalance, systemic inflammation, neuroimmune dysfunction, and cognitive decline (**Figure 3**). The latest findings suggest that microbiota modulation may represent a promising strategy to reduce age-related neurodegeneration.

**Figure 3:**
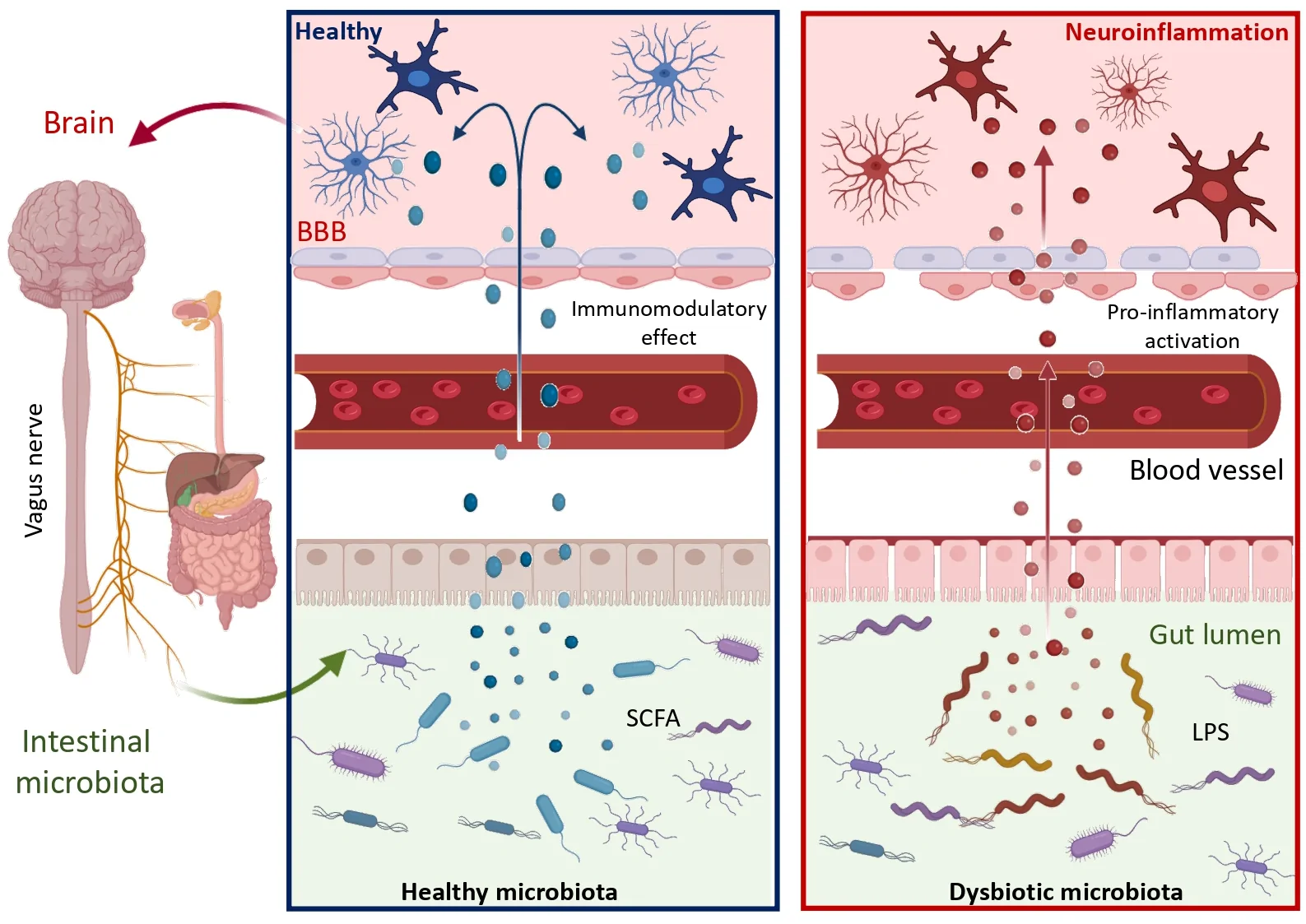
The microbiota–gut–brain axis in health and neuroinflammation. In healthy conditions, intestinal microbiota-derived metabolites, such as short-chain fatty acids (SCFA), exert immunomodulatory effects that maintain intestinal barrier integrity, regulate systemic inflammation, and support central nervous system homeostasis through signaling pathways involving the vagus nerve and circulation. In contrast, gut dysbiosis promotes the release of pro-inflammatory mediators and bacterial products such as lipopolysaccharides (LPS), leading to increased intestinal permeability, systemic inflammation, blood–brain barrier dysfunction, and activation of neuroinflammatory pathways. These processes contribute to microglial activation and neurodegeneration associated with Alzheimer’s disease.

## 5 Blood-brain barrier as a key player in controlling CNS inflammation

The BBB is a highly selective and dynamic interface that regulates the exchange of nutrients, metabolites, and signaling molecules between the systemic circulation and the CNS. Structurally, the BBB is formed by specialized endothelial cells connected by tight junctions and supported by pericytes, astrocytic end-feet, and extracellular matrix components, which together preserve barrier integrity and CNS homeostasis. Beyond its function as a physical barrier, the BBB actively participates in immune surveillance by restricting the entry of toxins, pathogens, and peripheral immune mediators into the brain parenchyma (Shay et al., 2023). Although essential for maintaining CNS homeostasis, the BBB also represents a major obstacle to the delivery of therapeutic agents for neurodegenerative disorders such as AD and PD (Li et al., 2026a). Importantly, several pathogens have evolved mechanisms to breach or disrupt the BBB, allowing access to the CNS through hematogenous routes. Pathogen infiltration is frequently associated with BBB dysfunction, which promotes neuroinflammation, immune cell recruitment, and neuronal damage (Wohlfert et al., 2017; Adamu et al., 2024). Once pathogens or pathogen-derived components reach the CNS, they activate innate immune responses and stimulate the release of pro-inflammatory mediators. Viral infections can induce cytokines such as TNF, IFN-β, IFN-γ, and IFN-λ, which weaken tight junctions and increase BBB permeability (Wang et al., 2004). Inflammatory signaling also promotes circulating immune cell recruitment and matrix metalloproteinase release, leading to extracellular matrix degradation and further disruption of BBB integrity (Persidsky et al., 2006). Moreover, some viruses directly alter endothelial cell function, thereby facilitating BBB breakdown and viral entry into the CNS (Kaur et al., 2023). Bacteria can also cross the BBB through multiple mechanisms, including direct endothelial infection, paracellular passage through intercellular junctions, and toxin-mediated barrier disruption (Jimenez-Munguia et al., 2021). For example, *Streptococcus pneumoniae* exploits adhesins such as RrgA and PspC to bind endothelial receptors including the polymeric immunoglobulin receptor and the platelet/endothelial cell adhesion molecule-1, thereby promoting bacterial entry and meningitis development (Iovino et al., 2017). *Neisseria meningitidis* uses type IV pili to form microcolonies on endothelial surfaces and activate intracellular signaling pathways that destabilize intercellular junctions. In addition, LPS from Gram-negative bacteria induces strong inflammatory responses that promote tight junction degradation and increase BBB permeability (Delbaz et al., 2020). In chronic infections, pathogens may also exploit the Trojan horse mechanism, whereby infected leukocytes cross the BBB and transport infectious agents into the CNS (Mcgavern and Kang, 2011). Once inside the brain, pathogens may disseminate through distinct routes and establish persistent low-level infections that maintain chronic neuroinflammation and progressive barrier dysfunction. For instance, *Toxoplasma gondii* infection can induce neuroinflammation and alter BBB integrity, with consequences for host behavior (Castano Barrios et al., 2021). Thus, the BBB should not be viewed only as a passive anatomical barrier, but as an active immunological interface that links peripheral infection, systemic inflammation, and CNS vulnerability. Sustained BBB dysfunction represents a critical event in infection-driven neurological damage. By facilitating pathogen entry and amplifying inflammatory responses, BBB disruption may contribute to the progression of neurodegenerative diseases and connect peripheral immune insults to long-term CNS alterations.

## 6 Controversies and limitations of the pathogen hypothesis

As extensively discussed in this paper, a substantial body of evidence supports the pathogen hypothesis of AD and its associated neuroinflammation. Indeed, this hypothesis accounts for the sustained microglial activation and neuroinflammation driven by infectious agents, providing a mechanistic driver for chronic immune cascades that precede overt clinical neurodegeneration. A major strength of this paradigm is the antimicrobial protection hypothesis, which frames Aβ not merely as a toxic byproduct, but as an innate immune peptide able to entrap invading microbes (Kumar et al., 2016). Furthermore, population-based epidemiological cohorts demonstrate that chronic neurotropic or systemic infections are associated with a significantly elevated long-term risk of dementia, whereas antiviral and antimicrobial treatments correlate with a reduced incidence of dementia in large observational studies (Livingston et al., 2024; Panza et al., 2019). However, the pathogen hypothesis remains highly controversial and faces several limitations. The primary criticism stems from the high prevalence of target microbes in the healthy elderly population: while pathogens such as HSV-1, *P.gingivalis*, and *C. pneumoniae* are often detected in AD brains, they are frequently identified in age-matched healthy control brains as well (Robinson et al., 2004). This raises fundamental questions as to whether microbial presence acts as a primary trigger of neurodegeneration or represents mere opportunistic colonization facilitated by age-related BBB breakdown and immunosenescence. Furthermore, literature findings exhibit marked heterogeneity, as different brain banks and patient cohorts frequently fail to consistently show specific viral or bacterial strains. These discrepancies are largely attributed to variations in detection sensitivities (e.g., highly sensitive PCR assays prone to contamination) and differences in post-mortem tissue preservation. Another crucial limitation arises from negative or inconclusive clinical trials: to date, interventions targeting infectious agents (e.g., small-molecule gingipain inhibitors against *P. gingivalis* or antiviral strategies like valacyclovir against HSV-1) have failed to demonstrate robust, reproducible cognitive improvements in Phase 2 or Phase 3 trials (Catumbela et al., 2023). Nonetheless, infections remain an undeniable driver of systemic and central inflammation. Given that neuroinflammation is a recognized hallmark of AD and a probable trigger of its pathogenesis, reframing pathogens not only as the sole primary cause, but as potent modifiers and drivers of neuroinflammation, may represent the most accurate approach.

## 7 Conclusion

AD is a multifactorial neurodegenerative disorder in which neuroinflammation plays a central and sustained role. While classical hallmarks such as Aβ deposition and tau pathology remain key features of the disease, increasing evidence supports the contribution of infectious agents as potential triggers and amplifiers of neuroinflammatory processes. A wide range of pathogens including viruses, bacteria, and fungi have been implicated in AD pathogenesis. These microorganisms may contribute through direct neuroinvasion or, more commonly, through indirect mechanisms involving systemic inflammation, immune activation, and disruption of the BBB. The concept of a pathogen driven contribution to AD is further supported by the antimicrobial properties of Aβ peptides, suggesting that their accumulation may initially represent a protective innate immune response that becomes disadvantageous when chronically activated. Similarly, the microbiota-gut-brain axis has emerged as a critical modulator of neuroinflammation, linking peripheral microbial dysbiosis to CNS dysfunction. Importantly, the convergence of multiple factors including aging, immune dysregulation, chronic infections, and barrier impairment likely determines individual susceptibility to neurodegeneration (**Figure 4**). The ability of pathogens to induce sustained neuroinflammatory responses highlights potential therapeutic opportunities. Strategies targeting infections, modulating immune responses, restoring microbiota balance, or preserving BBB integrity may offer novel approaches to prevent or slow AD progression. Despite significant advances, a causal relationship between specific pathogens and AD has not been definitively established, and further longitudinal and mechanistic studies are required to clarify their role. In conclusion, understanding the interplay between infectious agents and neuroinflammation may provide new insights into AD pathogenesis and open the way for innovative, multi-target therapeutic strategies aimed at reducing the burden of this devastating disease.

**Figure 4:**
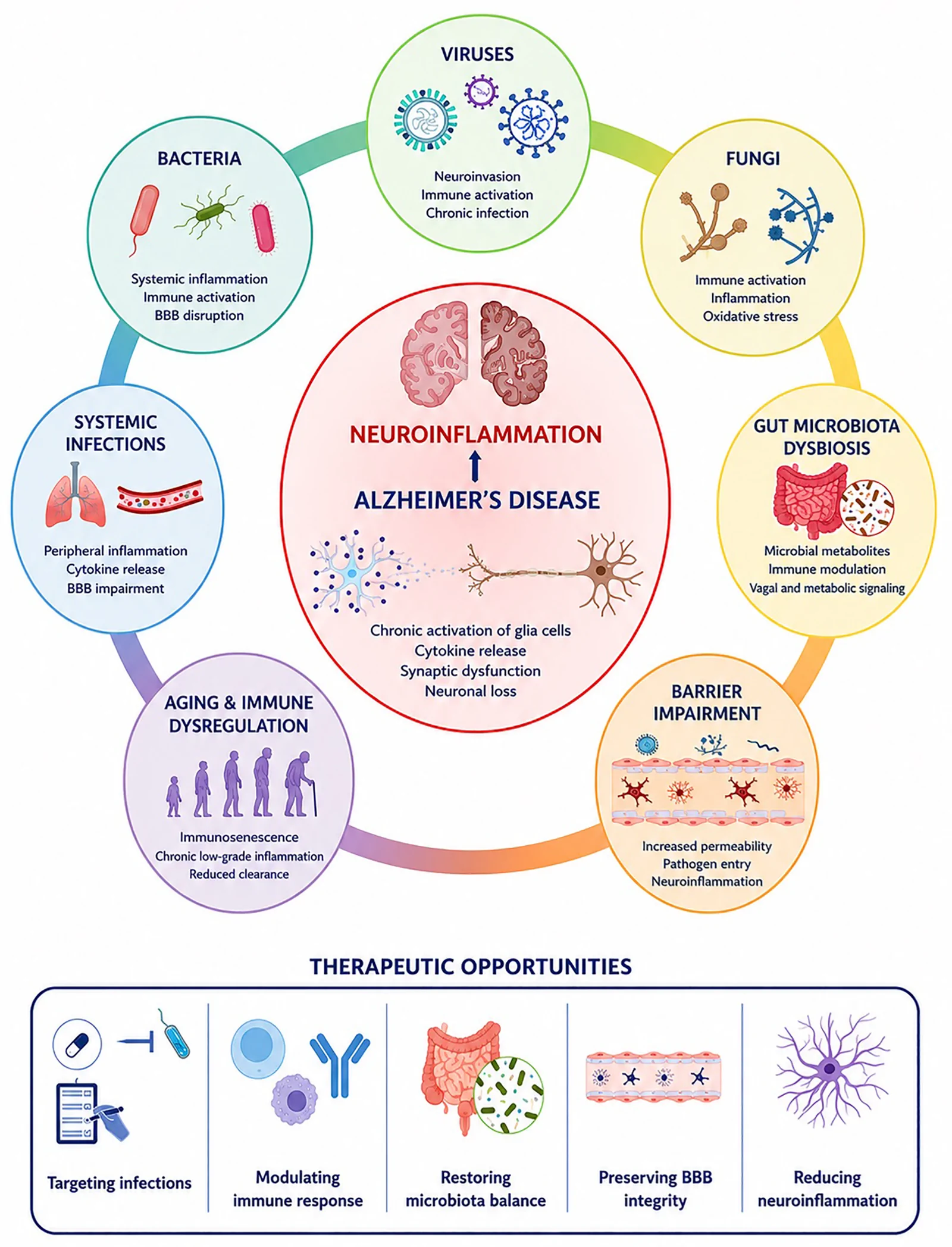
Interconnected pathogenic mechanisms promoting chronic neuroinflammation in Alzheimer’s disease. Infectious agents (viruses, bacteria, and fungi), gut microbiota dysbiosis, systemic infections, aging-associated immune dysregulation, and barrier impairment converge to promote chronic neuroinflammation, a central driver of Alzheimer’s disease, by glial activation, cytokine release, synaptic dysfunction, and neuronal loss. Collectively, these interconnected mechanisms highlight potential therapeutic strategies targeting infections, immune responses, microbiota homeostasis, BBB integrity, and chronic neuroinflammation.
